# Massive Intestinal Bleeding in an Adult with IgA Vasculitis Treated with Intravenous Immunoglobulin

**DOI:** 10.1155/2022/9694911

**Published:** 2022-06-14

**Authors:** Ibrahim Nassereddin, Ariel Kenig, Yuval Ishay, Hillel Lehmann, Noa Hurvitz, Narmine Elkhateeb, Ram Gelman, Yael Ratz, Inon Sarig, Ido Burstain, Stephanie Benshushan, Fadi Kharouf

**Affiliations:** ^1^Department of Medicine, Hadassah Medical Center, The Faculty of Medicine, Hebrew University, Jerusalem, Israel; ^2^The Institute of Gastroenterology and Liver Diseases, Hadassah Medical Center, The Faculty of Medicine, Hebrew University, Jerusalem, Israel; ^3^The Nephrology Unit, Hadassah Medical Center, The Faculty of Medicine, Hebrew University, Jerusalem, Israel; ^4^Department of Pathology, Hadassah Medical Center, The Faculty of Medicine, Hebrew University, Jerusalem, Israel; ^5^The Rheumatology Unit, Hadassah Medical Center, The Faculty of Medicine, Hebrew University, Jerusalem, Israel

## Abstract

We report the case of a 29-year-old adult presenting with severe IgA vasculitis, with cutaneous, urologic, and renal manifestations. The late appearance of severe gastrointestinal bleeding dominated the clinical picture, necessitating the administration of tens of units of packed cells and the augmentation of the immunosuppressive protocol. It was not until therapy with intravenous immunoglobulin (IVIG) was introduced that the massive bleeding was controlled. We herein discuss the patient's presentation, the gastrointestinal manifestations of IgA vasculitis, the recommended treatments, and the existent evidence about IVIG therapy.

## 1. Introduction

Life-threatening gastrointestinal (GI) bleeding is a rare occurrence in adults with IgA vasculitis (formerly known as Henoch–Schönlein purpura) [1]. Evidence-based therapeutic approaches for such manifestations are still lacking. Herein, we report the case of a 29-year-old male patient with a new diagnosis of IgA vasculitis, who suffered from hemorrhagic shock, secondary to severe GI bleeding, complicating the disease course. Based on sporadic evidence from the pediatric literature [2], he received intravenous immunoglobulin (IVIG), as an add-on therapy, leading to rapid control of the massive bleeding event.

## 2. Case Presentation

A 29-year-old male patient was admitted to our tertiary care center for a two-week history of fever, watery diarrhea, oliguria, and lower limb and scrotal edema. His medical record was remarkable for uncontrolled insulin-dependent diabetes mellitus, with secondary target organ insults, including severe retinopathy, chronic renal failure, sensorimotor peripheral neuropathy, and a diabetic ulcer in his right foot.

In the emergency department, the patient was tachycardic (106 beats/min), with elevated blood pressure (165/85 mmHg) and low-grade fever (38°C); oxygen saturation in room air was normal. Physical examination revealed severe bilateral leg pitting edema and a markedly swollen scrotum. Minor purulent discharge was present in the right leg ulcer. Initial laboratory analysis was notable for deteriorating renal function, severe normocytic anemia, and elevated inflammatory markers (see Table 1). Urinalysis was positive for blood and protein; urinary sediment displayed granular casts, calcium oxalate crystals, and a few erythrocytes, some of which were dysmorphic. Chest X-ray showed signs of pulmonary congestion, and abdominal ultrasonography demonstrated atrophic kidneys and mild ascites. A total body computed tomography (CT) scan revealed anasarca, with mild right-sided hydronephrosis.

The patient was admitted to the medical ward, and hemodialysis was initiated. Microbiologic workup, including blood, urine, and stool cultures was negative. Viral serologies for human immunodeficiency virus, hepatitis B virus, and hepatitis C virus were negative. Likewise, antinuclear antibody, perinuclear anti-neutrophilic cytoplasmic antibody (p-ANCA), cytoplasmic-ANCA (c-ANCA), and cryoglobulin were negative. Complement proteins were unremarkable, and IgA levels were elevated (460 mg/dL; normal: 70–400). Transthoracic echocardiography was normal, with no vegetations seen.

While hospitalized, a purpuric rash started to develop on the patient's lower limbs, being more prominent on the extensor surfaces and extending proximally to the buttocks. In light of the clinical picture of renal impairment, watery diarrhea, severe scrotal edema, and purpuric rash, small vessel vasculitis was considered. Oral corticosteroid therapy (60 mg/day of prednisone) was started, besides empiric antibiotic therapy for a suspected soft tissue foot infection.

**Figure 1:**
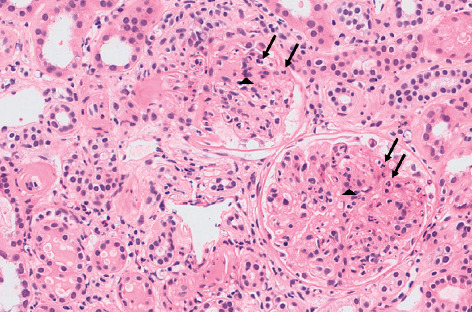
Renal biopsy with glomerular mesangial expansion and mesangial hypercellularity, including segmental neutrophil influx (arrows and arrowheads) (H&E, original magnification, ×200). H&E: hematoxylin and eosin.

**Figure 2:**
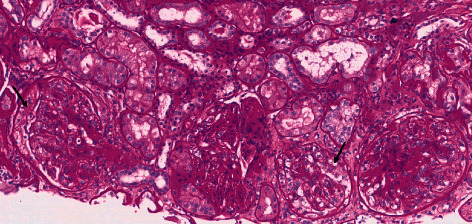
Early cellular crescents (arrows) are seen (PAS stain, original magnification, ×160). PAS: Periodic acid–Schiff.

A skin biopsy was performed, showing leukocytoclastic vasculitis, with IgA-C3 deposition on immunofluorescence. Besides, renal biopsy was carried out, revealing glomerulonephritis, with mesangial expansion, neutrophil influx, and cellular crescents (see Figure 1 and 2). Immunopathology displayed mesangial IgA deposition.

A diagnosis of IgA vasculitis was assigned, and considering the severe acute renal deterioration, pulses of methylprednisolone were started (500 mg/day for 3 days). 500 mg of intravenous (IV) cyclophosphamide (CYC) was also administered for induction.

One day following the administration of CYC, under high-dose IV methylprednisolone therapy (35 mg twice/day), severe bloody diarrhea appeared. At the same time, no major change in the purpuric rash and scrotal edema was observed. A broad radiographic investigation, including abdominal CT angiography, gastroscopy, colonoscopy, radionuclide scanning, and conventional angiography failed to localize the source of bleeding. Hemoglobin declined to as low as 5.6 g/dL (with a normal platelet count and coagulation profile), despite the administration of packed cells (PCs); the rectal bleeding was massive and the patient developed hemorrhagic shock. He was immediately transferred to the intensive care unit, receiving IV fluids and tens of units of PCs. IV methylprednisolone was renewed at a dose of 250 mg/day for 3 days, assuming a vasculitic etiology. At this stage, a multidisciplinary meeting was carried out, consisting of rheumatology, gastroenterology, nephrology, and radiology staffs. Upon revision of the patient's imaging studies, areas of severe jejunal edema were present, thus jejunal vasculitis was suspected.

Based on small series in the literature, IVIG therapy was started, with the patient receiving 120 grams over a period of 5 days. A second dose of IV CYC 500 mg was also given, 14 days after the first one. By the end of the IVIG therapy, the bleeding, which necessitated 40 units of PCs stopped, and the patient was transferred to the medical ward on prednisone 60 mg/day. Continuation of steroid therapy, besides hemodialysis, led to the resolution of the severe volume overload and scrotal edema, besides the total disappearance of the purpuric rash. Renal function did not recover, and the patient remained dialysis-dependent.

After prolonged hospitalization, the patient was discharged. It was planned to continue induction therapy with rituximab, but a severe infectious event in the right foot diabetic ulcer postponed therapy. He had to undergo a below-knee amputation three months post-discharge. Considering his predisposition to infections, and after accumulating evidence of irreversible renal failure and cessation of the GI bleeding event, it was decided to carry on a gradual steroid-tapering regimen, with no additional induction protocol.

## 3. Discussion

We have herein reported a unique case of adult-onset IgA vasculitis, manifesting as typical rash, scrotal edema, life-threatening GI bleeding, and end-stage renal disease. The rarity of the case and lack of tested therapies for this scenario necessitated borrowing treatment strategies from the pediatric literature.

While childhood-onset IgA vasculitis tends to follow a benign course, the disease prognosis in adults is worse, particularly due to the frequency and severity of renal involvement. Kidney disease is detected in 45% to 85% of cases of adult-onset IgA vasculitis, with a 30% risk for progression to renal insufficiency [3]. Patients older than 20 years of age, with GI involvement at onset and persistent skin eruption, are more likely to progress to renal disease [4, 5].

Genitourinary involvement is a known feature of IgA vasculitis. In a retrospective study of 122 Korean boys, the scrotal disease was present in 22% of the patients, with scrotal swelling appearing in 23% of the patients and scrotal pain and tenderness in 18% [6]. In another cross-sectional retrospective study, scrotal signs and symptoms seemed to improve after prompt use of corticosteroids and were associated with a low frequency of elevated IgA serum levels [7].

In adults, the GI tract is affected in approximately half of the cases of IgA vasculitis. Most cases present with colicky abdominal pain, and only a third of patients experience GI bleeding. When the disease proves fatal, death is usually attributed to either GI or renal causes [8, 9]. In a study by Hocevar et al., generalized purpura, elevated pretreatment neutrophil to lymphocyte ratio and elevated serum IgA predicted both GI and renal complications [10]. This may resonate with the association of GI manifestations with worse renal outcomes [11].

Imaging studies of patients with GI manifestations usually show intestinal wall thickening. On endoscopy, mucosal ulcerations, erythema, and purpura are the typical lesions, most frequently involving the duodenum and small intestine. Notably, biopsied GI lesions often show inflammatory infiltrates but no hallmark leukocytoclastic vasculitis [1].

The treatment of adult-onset IgA vasculitis is controversial. It is based, depending on disease severity, on corticosteroid therapy and immunosuppressive agents, including cyclophosphamide, azathioprine, mycophenolate, and cyclosporine. Rituximab has also been shown to be an effective treatment in some studies [12, 13]. In patients with refractory GI manifestations, as in our case, there is a paucity of data to guide clinicians. The literature regarding advanced treatment lines is scarce in children and almost nonexistent in adults. Corticosteroids, CYC, and plasmapheresis are the leading therapeutic options based on anecdotal reports [1, 11].

In 2016, Cherqaoui et al. reported eight children with intractable abdominal pain, GI bleeding, or protein-losing enteropathy who failed to respond to steroids and were treated with IVIG. Six patients responded promptly to the first course, and an additional course was required in two [2]. This was further substantiated by reports of children with GI-predominant disease responding to IVIG [14], and a response to IVIG demonstrated in adults with renal involvement [15, 16].

While there is no definite evidence that the impressive recovery can be directly attributed to the IVIG therapy in our case, the fact that the GI bleeding originally developed under high-dose corticosteroids and IV CYC (given for the other disease manifestations), besides the temporal association between the administration of IVIG and clinical improvement, both support the belief that it was the main therapeutic game changer.

## 4. Conclusion

Life-threatening presentations of IgA vasculitis in the adult are infrequent, but GI manifestations count among them when they occur. Solid evidence for treatment protocols is invalid, especially in cases resistant to corticosteroids and CYC. In our case, we were sufficiently galvanized by reports of IVIG efficacy, the refractoriness of our patient to treatment, and the lack of further immediate therapeutic options to attempt using IVIG. We are glad to report our short-term success in terminating the severe GI bleeding in our patient. We believe IVIG may be considered in refractory cases of adult-onset IgA vasculitis with severe GI manifestations.

## Figures and Tables

**Table 1:** Laboratory results during admission and massive GI bleeding.

	Normal values	Admission	Major GI bleeding
Creatinine (mmol/L)	62–115	**517**	428^a^
Urea (mmol/L)	3.2–8.2	**32.7**	**13.0**
Albumin (g/L)	32–48	**27**	**18**
Alanine aminotransferase (U/L)	10–49	12	<7
Alkaline phosphatase (U/L)	46–116	74	65
Total bilirubin (*µ*mol/L)	5–21	12.3	4.9
Hemoglobin (g/dL)	13.9–17.7	**7.8**	5.6
White blood cells (×109 cells/L)	3.79–10.33	9.2	5.3
Neutrophils (×109 cells/L)	1.78–7	6.7	3.8
Platelets (×109 cells/L)	166–389	162.0	288.0
C-reactive protein (mg/dL)	0–0.5	**7.84**	**4.11**
Prothrombin time (%)	63.8–127.7	83.6	80.6
PTT (seconds)	22.4–36.3	32.6	26.2
INR	0.9–1.2	1.09	1.11

GI, gastrointestinal; PTT, partial thromboplastin time; INR, international normalized ratio. ^a^Patient was on hemodialysis treatment. Abnormal results appear in bold.

## Data Availability

The data can be made available upon request from the corresponding author.

## Abbreviations

GI: Gastrointestinal
IVIG: Intravenous immunoglobulin
CT: Computed tomography
ANCA: Antineutrophil cytoplasmatic antibodies
IV: Intravenous
CYC: Cyclophosphamide
PC: Packed cells.
